# Supplementary material for: Biologics and Small Molecule Inhibitors for Treating Hidradenitis Suppurativa: A Systematic Review and Meta-Analysis
Source: Biomedicines. 2022 Jun 2;10(6):1303. doi: 10.3390/biomedicines10061303 (PMC9220298; doi:10.3390/biomedicines10061303)
Supplement: Supplementary file 1 [file biomedicines-10-01303-s001.zip › biomedicines-1759983-supplementary.pdf]

biomedicines

MDPI

Table S1. *Search strategy Cochrane library, Embase, and Medline (Ovid)* 1

**Cochrane library**	
#1	MeSH descriptor: [Hidradenitis Suppurativa] explode all trees
#2	acne invers*
#3	invers* acne
#4	hidradeniti* suppurativ*
#5	suppurativ* hidradeniti*
#6	velpeau* disease
#7	verneuil* disease
#8	#1 or #2 or #3 or #4 or #5 or #6 or #7
**Embase**	
#1	'acne invers*':ab,ti
#2	'invers* acne':ab,ti
#3	'hidradeniti* suppurativ*':ab,ti
#4	'suppurativ* hidradeniti*':ab,ti
#5	'velpeau* disease':ab,ti
#6	'verneuil* disease':ab,ti
#7	'suppurative hidradenitis'/exp OR 'hidradenitis suppurativa' OR 'suppurativa, hidradenitis' OR 'suppurative hidradenitis'
#8	#1 OR #2 OR #3 OR #4 OR #5 OR #6 OR #7
#9	'crossover procedure':lnk OR 'cross over clinical study' OR 'cross over clinical trial' OR 'cross over comparison' OR 'cross over design' OR 'cross over method' OR 'cross over procedure' OR 'cross over study' OR 'cross over test' OR 'cross over trial' OR 'cross-over studies' OR 'crossover clinical study' OR 'crossover clinical trial' OR 'crossover comparison' OR 'crossover design' OR 'crossover method' OR 'crossover procedure' OR 'crossover study' OR 'crossover test' OR 'crossover trial' OR 'double blind cross over study' OR 'double blind crossover study' OR 'procedure, crossover'
#10	'double blind procedure':lnk OR 'double blind clinical trial' OR 'double blind comparison' OR 'double blind design' OR 'double blind procedure' OR 'double blind studies' OR 'double blind study' OR 'double blind test' OR 'double blind trial' OR 'double masked clinical study' OR 'double masked clinical trial' OR 'double masked comparison' OR 'double masked design' OR 'double masked method' OR 'double masked procedure' OR 'double masked study' OR 'double masked test' OR 'double masked trial' OR 'double-blind clinical study' OR 'double-blind method'
#11	'single blind procedure':lnk OR 'procedure, single blind' OR 'single blind clinical study' OR 'single blind clinical trial' OR 'single blind comparison' OR 'single blind design' OR 'single blind procedure' OR 'single blind studies' OR 'single blind study' OR 'single blind test' OR 'single blind trial' OR 'single masked clinical study' OR 'single masked clinical trial' OR 'single masked comparison' OR 'single masked design' OR 'single masked method' OR 'single masked procedure' OR 'single masked study' OR 'single masked test' OR 'single masked trial' OR 'single-blind method' OR 'study, single blind'
#12	crossover?:ti,ab,kw,de OR 'cross over?':ti,ab,kw,de
#13	PBO*:ti,ab,kw,de
#14	doubl*:ti,ab,kw,de AND near:ti,ab,kw,de AND blind*:ti,ab,kw,de

#15	allocat*:ti,ab,kw,de
#16	trial:ti
#17	'randomized controlled trial':lnk OR 'controlled trial, randomized' OR 'randomised controlled study' OR 'randomised controlled trial' OR 'randomized controlled study' OR 'randomized controlled trial' OR 'trial, randomized controlled'
#18	random*:ti,ab,kw,de
#19	#9 OR #10 OR #11 OR #12 OR #13 OR #14 OR #15 OR #16 OR #17 OR #18
#20	('animal'/de OR 'nonhuman'/de OR 'animal experiment'/de OR 'animal experiment' OR 'animal experimentation' OR 'animal physical conditioning' OR 'animal studies' OR 'animal study' OR 'animal trial' OR 'experiment, animal' OR 'physical conditioning, animal') AND ('human'/de OR 'human' OR 'humans')
#21	'animal'/de OR 'nonhuman'/de OR 'animal experiment'/de OR 'animal experiment' OR 'animal experimentation' OR 'animal physical conditioning' OR 'animal studies' OR 'animal study' OR 'animal trial' OR 'experiment, animal' OR 'physical conditioning, animal'
#22	#21 NOT #20
#23	#19 NOT #22
#24	#8 AND #23
#25	#24 AND [embase]/lim NOT ([embase]/lim AND [medline]/lim)
**Medline (Ovid)**	
1	exp Hidradenitis Suppurativa/
2	acne invers$1.ti,ab.
3	invers$ acne.ti,ab.
4	hidradeniti$ suppurativ$.ti,ab.
5	suppurativ$ hidradeniti$.ti,ab.
6	velpeau$ disease.ti,ab.
7	verneuil$ disease.ti,ab.
8	or/1-7
9	randomized controlled trial.pt.
10	controlled clinical trial.pt.
11	randomized.ab.
12	PBO.ab.
13	clinical trials as topic.sh.
14	randomly.ab.
15	trial.ti.
16	9 or 10 or 11 or 12 or 13 or 14 or 15
17	exp animals/ not humans.sh.
18	16 not 17
19	8 and 18
[Lines 9-18: Cochrane Highly Sensitive Search Strategy for identifying randomized trials in MEDLINE: sensitivity- and precision-maximizing	

Table S2. *Summary of Included Randomized Controlled Trials*

Author, year, country	Registration	Period / Primary endpoint	Severity	Intervention	Dose	Mean age (SD)	Sex (M/F)	Tool	Result
Alavi 2022, Canda, Denmark, Germany	NCT 03607487	NA / 8 weeks	Hurley II/III	INCB054707	30mg QD	41(11.53)	2/7	HiSCR	INCB: 5/9(55.6%), 5/9(55.6%), 7/8(87.5%); PBO: 4/7(57.1%)
					60mg QD	42.2 (11.96)	1/8	IHS4	Mean change: INCB: -9.4(6.8), -21.4(21.4), -16.1(22.9); PBO: -10.7(21.0)
					90mg QD	42.8 (14.62)	3/5	MSS	Mean change: INCB: -41.9 (36.84), -59.2 (48.48) , -54.6 (55.42); PBO: -36.4(35.83)
								HiSQoL	Mean change: INCB: -2.2(2.2), -1.4(1.4), -3.1(3.3); PBO: 0.3(2.8)
				PBO	X	40.3 (16.70)	1/8	DLQI	Mean chabnge: INCB: -7.2(7.1), -4.2(4.2), -5.8(4.7), 0.9(6.5)
								NRS for pain	Mean change: INCB: -2.2(2.18), -1.4(1.44), -3.1 (3.28); PBO: 0.3(2.77)
Bechara 2021, Germany	NCT 02808975 (SHARPS)	Jul. 2016 – Oct. 2019 / 12 weeks	Hurley II/III	ADA	160mg Wk0→ 80mg Wk 2→ 40mg QW	38.5(11.7)	52/51	HiSCR	ADA:49/103(47.6%), PBO:35/103(34%)
								DLQI	Mean change: ADA: -4.8; PBO: -1.3
				PBO	X	36.8(10.8)	48/55	HSIA	Mean change: ADA: -1.83; PBO: -0.37
								HSSA	Mean change: ADA: -2.03; PBO: -0.53
								PtGA	Mean change: ADA -2.0, PBO: -0.5

Table S2. *Cont.*

Author, year, country	Registration	Period / Primary endpoint	Severity	Intervention	Dose	Mean age (SD)	Sex (M/F)	Tool	Result
Jassen Research & Development LLC 2021, USA, Canada, Europe	NCT 03628924	Sep. 2018 –May 2020 / 16 weeks	Hurley II/III	Guselkumab	200mg SC	39 (12.3)	27/32	HiSCR	Guselkumba: 30/59(50.8%), 27/60(45%); PBO: 24/62(38.7%)
								DLQI	Mean change: Guselkumab: -3.4 (6.81), -2.5(6.13); PBO: -0.7 (5.17)
					1200mg IV to 200mg SC	37.2 (10.9)	15/45	HSSD	Mean change: Guselkumab: -1.71 (2.325), -0.82(2.148); PBO: -0.19 (2.124)
				PBO				HS-IGA 0/1	Guselkumab: 17/59(28.8%), 14/60(23.3%) ; PBO: 15/62(24.2%)
					PBO	38.2 (11.6)	24/38	HADS	Mean change:Anxiety/Depression: Guselkumab: 0.0(2.82)/-0.6(2.73), -0.3(2.56)/ -0.5 (2.69); PBO: 0.0(2.60)/0.2(2.48)
Glatt 2021, USA, Europe, Australia, Russia	NCT 03248531	Sep. 2017 –Feb. 2019 /12 weeks	Hurley II/III	Bimekizumab	640mg W0→ 320mg Q2W	37.4 (11.9)	16/30	HiSCR	Bimekizumab: 25/40 (63%), ADA: 12/18(67%), PBO: 5/18(28%)
				ADA	160mg W0 →80mgW2 →40mg QW	31.1 (9.4)	4/17	IHS4	Bimekizumab: 16.0(18), ADA: 16.5, PBO: 40.2(32.6)
								DLQI 0/1	Bimekizumab: 14/39 (36%), ADA: 3/21(14%), PBO:0
				PBO	X	40.7 (12.8)	7/14	PtGA	≥30% and ≥1 unit reduction: Bimekizumab: 27/42(64%), ADA: 9/18(50%), PBO: 7/19(37%)

Table S2. *Cont.*

Author, year, country	Registration	Period / Primary endpoint	Severity	Intervention	Dose	Mean age (SD)	Sex (M/F)	Tool	Result
Giamarellos 2021, USA, Canada, Europe	NCT 03487276	NA / 16 weeks	Hurley II/III	IFX-1	400mg Q4W	39	18/16	HiSCR	IFX-1: 12/30 (40%), 17/33 (51.5%), 12/31 (38.7%), 15/33 (45.5%); PBO:16/34 (47.1)
					800mg Q4W	35	17/18	MSS	Absolute change (D1-D309):<br>IFX-1: -17.5 (48.43), -29.4 (32.35), -22.9(52), -35.2(101.9); PBO: -16.4 (30.78)
					800mg Q2W	37	16/20	DLQI	Absolute change (D1-D309):<br>IFX-1: 0.6(6.39), -2.6(7.19), -5.0(5.90), -2.4 (5.24); PBO: -1.5(6.11)
					1200mg Q2W	33.5	13/23	PtGA	Absolute change (D1-D309):<br>IFX-1: -0.1(2.68), -0.7(2.37), -1.5 (2.92), -1.8 (3.03); PBO: -1.2 (2.82)
				PBO	X	34.5	15/21	IHS4	Mean change:<br>IFX-1(1200mg) 51.5%; PBO: 19.8%
Kirby 2021, USA	NCT 03852472	NA / 12 weeks	Hurley II/III	Avacopan	10mg BID	X	134	HiSCR	Avacopan: 30/134 (22.4%), 47/134 (35.1%); PBO: 40/130 (30.8%)
					30mg BID		134		
				PBO	PBO		130		
Kimball 2020, USA, Europe	NCT 02421172	NA / 16 weeks	Hurley II/III	CJM112	300mg QW*5→ bi-weekly*5	36(9.8)	11/22	HS-PGA	CJM112:10/31(32.3%), PBO: 4/32 (12.5%)
				PBO	X	39(10.9)	11/22		

Table S2. *Cont.*

Author, year, country	Registration	Period / Primary endpoint	Severity	Intervention	Dose	Mean age (SD)	Sex (M/F)	Tool	Result
Vossen 2019, Netherlands	EudraCT 2016-000859-27	Feb. 2017 - Aug. 2017 / 16 weeks	HS-PGA moderate	Apremilast	10mg on D1 → increase of 10 mg/d → 30mg BID	35.7 (13)	3 /12	HiSCR	APR: 8/15(53.3%), PBO: 0
								DLQI	Mean change: APR: -2.3; PBO: +4.2
				PBO	X	33.4 (8.2)	0/5	NRS	Mean change: Pain/itch/disease burden: APR: -0.8 / -1.1/ -1.0; PBO: +2.2/+2.4/+0.9
Kanni 2018, Greece	NCT 02643654	Dec. 2015 - Jan.2017 / 12 weeks	Hurley II/III	MABp1	7.5mg/kg Q2W	46.6(15.1)	7/3	HiSCR	MABp1: 6/10 (60%), PBO: 1/10 (10%)
								MSS	p = 0.879
				PBO	X	49.3(9.8)	6/4	VAS	p = 0.091
								VAS for pain	p = 0.255
Tzanetakou 2016, Greece	NCT 01558375	Mar. 2012 - Feb. 2014 / 12 weeks	Hurley II/III	Anakinra	100mg QD	42.3(13.8)	5/4	HiSCR	Anakinra: 7/9(78%), PBO: 3/10(30%)
								SS	p > .05
				PBO	X	36.0(11.3)	5/5	DLQI	p > .05
								VAS	p > .05 (for pain: p > .05)

Table S2. *Cont.*

Author, year, country	Registration	Period / Primary endpoint	Severity	Intervention	Dose	Mean age (SD)	Sex (M/F)	Tool	Result
Kimball 2016, USA, Europe, Australia, Puerto Rico, Turkey	NCT 01468207 PIONEER I	Nov. 2011 – Jan. 2014 / 12 weeks	Hurley II/III	ADA	160mg W0 →80mgW2 →40mg QW	36.2 (10.8)	62/91	HiSCR	ADA: 64/153(41.8%); PBO:40/154(26%)
								MSS	ADA: 125.8; PBO: 130.5<br>Mean change: ADA: -24.4; PBO: -15.7
				PBO	X	37.8 (11.3)	49/ 105	DLQI	0/1: ADA: 10/153(6.5%), PBO: 2/154(1.3%);<br>Mean change: ADA: -5.4, PBO: -2.9
								PtGA	Mean change: ADA: -1.3, PBO: -0.7
								WPAI-SHP	Mean change: Overall work/activity impairment:<br>ADA: -13.4/-14; PBO: -9.9/-8.3
								TSQM	Mean change: Global satisfaction: ADA 17; PBO: 8.4
								SF-36	Mean change: Physical/Mental component summary:<br>ADA: 4.2/2.3; PBO: 1.5/1.3
								HADS	Mean change: Anxiety/Depression:<br>ADA: -1.4/-1.5; PBO: -0.8/-1.0
Kimball 2016, USA, Europe, Australia, Puerto Rico, Turkey	NCT 01468233 PIONEER II	Dec. 2011 –Apr. 2014 / 12 weeks	Hurley II/III	ADA	160mg W0 →80mgW2 →40mg QW	34.9 (10.0)	55/ 108	HiSCR	ADA:96/163(58.9%); PBO: 45/163(27.6%)
								MSS	ADA: 81.4; PBO: 115.2<br>Mean chabge: ADA: -28.9; PBO: -9.5
				PBO	X	36.1 (12.2)	50/ 113	DLQI	0/1: ADA: 13/163(8%), PBO: 4/163(2.5%);<br>Mean change: ADA: -5.1, PBO: -2.3
								PtGA	Mean change: ADA: -2.3, PBO: -0.7
								WPAI-SHP	Mean change: Overall work/activity impairment:<br>ADA: -13.4/-14.5; PBO: -5.9/-7.2
								TSQM	Mean change: Global satisfaction: ADA: 22.7; PBO: 8.6
								EQ-5D	Mean change: ADA: 0.1; PBO: 0

Table S2. *Cont.*

Author, year, country	Registration	Period / Primary endpoint	Severity	Intervention	Dose	Mean age (SD)	Sex (M/F)	Tool	Result
Kimball 2012, USA, Europe	NCT 00918255	Apr. 2009 – Nov. 2010 / 16 weeks	HS-PGA Moderate/ Severe	ADA (ew)	160mg D0→ 80mg W2→ 40mg QW	35.1 (10.7)	15/ 36	HiSCR	ADAew/eow: 24/44% (54.5%), ADAeow: 15/45 (33.3%), PBO: 11/43 (25.6%) (post hoc)
								HS-PGA	ADAew: 9/51(17.6%), ADAeow: 5/52(9.6%), PBO: 2/51(3.9%)
				ADA (eow)	80mg W0→ 40mg Q2W	36.1 (12.5)	14/ 38	MSS	Mean Change: ADAew: -40.2(9.8), ADAeow: -32(9.5), PBO: 17.2(9.8)
								DLQI	Mean Change: ADAew: -6.3(0.9), ADAeow: -3.2(0.8), PBO: -2.3(0.9)
				PBO	X	37.8 (12.1)	15/ 36	VAS (pain)	≥30% and 10-mm reduction: ADAew: 23/48(47.9%), ADAeow: 17/47(36.2%), PBO: 13/48(27.1%)
Miller 2011, Denmark	NA	2007 – Jul. 2010 / 12 weeks	Hurley II/III	ADA	80mg W0→ 40mg Q2W	38.7	3/ 12	SS	Mean change: ADA: -11.27, PBO: 5.83
								Hurley	Mean change: ADA: -0.13, PBO: 0
				PBO	X	40.2	1/5	DLQI	Mean change: ADA: -3.67, PBO: 1.0
								VAS	Mean change: ADA: -13.4, PBO: 3.17
Adams 2010, USA	NCT 00949546	NA / 12 weeks	Moderate to severe	Etanercept	50mg BIW	40	4/6	PGA	p > .99
				PBO	X	36.7	3/7	DLQI	p = .12
Grant 2010, USA	NCT 00795574	NA / 8 weeks	HSSI moderate / severe	Infliximab	5mg/kg at W0,2,6	34 (13.44)	3/ 12	HSSI	≥50% decrease: p=.092
								DLQI	Mean change: infliximab: -10, PBO: -1.6
				PBO	X	33.2 (11.42)	9/ 14	PGA	Infliximab: 1.8, PBO: 4.7
								VAS	Mean change: infliximab: -39.8, PBO: -0.6

ADA = adalimumab; BID = twice daily; BIW = twice weekly; DLQI= Dermatology life quality index; EW= Every week; Eow = Every other week; HADS= Hospital anxiety and depression scale; HiSCR= Hidradenitis suppurativa clinical response; HiSQoL = Hidradenitis Suppurativa Quality of Life Score; HSIA= Hidradenitis suppurativa impact assessment; HS-IGA= Hidradenitis suppurativa-investigator's global assessment; HS-PGA= Hidradenitis suppurativa physician global assessment; HSSA= Hidradenitis suppurativa symptom assessment; HSSD= Hidradenitis suppurativa symptom diary; HSSI= Hidradenitis suppurativa severity index; IHS4= Hidradenitis suppurativa severity score system; MSS= Modified Sartorius score; NRS= Numeric rating scale; PBO = placebo; PGA= Physician global assessment; PtGA= Patients' global assessment; QD = everyday; Q2W = every other week; Q4W = every four week; QW: every week; SF-36=

Short form-36 health status survey; SS= Sartotius score; TSQM= Treatment satisfaction questionnaire for medication; VAS= Visual analogue scale, W = Week, WPAI-SHP: Work productivity and activity impairment questionnaire: specific health problem
